# Model construction and thrombolytic treatment of rat portal vein thrombosis

**DOI:** 10.1371/journal.pone.0308178

**Published:** 2024-08-02

**Authors:** Zixiang Wang, Chenguang Su, Zheng Liao, Zixin Li, Jianli Wang, Shijie Fu, Jian Li, Jinlong Liu

**Affiliations:** 1 Department of Hepatobiliary Surgery, Affiliated Hospital of Chengde Medical College, Chengde, Hebei Province, China; 2 Department of Pathology, Affiliated Hospital of Chengde Medical College, Chengde, Hebei Province, China; 3 Department of Hand and Foot Surgery, Affiliated Hospital of Chengde Medical College, Chengde, Hebei Province, China; 4 Hebei Key Laboratory of Panvascular Diseases, Chengde, China; Tokai University School of Medicine: Tokai Daigaku Igakubu Daigakuin Igaku Kenkyuka, JAPAN

## Abstract

**Objective:**

To construct a stable rat portal vein thrombosis (PVT) model and explore the time window of urokinase thrombolytic therapy on this basis.

**Methods:**

Constructing a rat PVT model by combining anhydrous ethanol disruption of portal endothelium with stasis of blood flow. Forty-eight rats after PVT modeling were divided into control group and experimental group, with 24 rats in each group. The experimental and control groups were given urokinase treatment and saline tail vein injection, respectively. The two groups of rats were observed and compared for PVT formation at 1, 3 and 5 days after modeling, respectively.

**Results:**

A stable rat PVT model was successfully constructed. No significant differences were found in PVT length, portal vein wet weight, and percentage of luminal occlusion area in the control rats at 1, 3, and 5 days after successful modeling (P > 0.05). Compared with control rats 1 day after modeling, the percentage of non-organized thrombus luminal area was significantly decreased (P < 0.0001), and the percentage of organized thrombus luminal area was significantly increased (P < 0.0001) in the PVTs of control rats at 3 and 5 days after modeling. After thrombolytic treatment with urokinase, plasma fibrinogen (FBG) levels were significantly decreased in the experimental group of rats compared with the control group (P < 0.0001), and plasma D-dimer (D2D) levels were significantly increased in the experimental group of rats compared with the control group (P < 0.0001). In addition, we observed prolongation of prothrombin time (PT) in the experimental group at 1, 3 and 5 days after modeling compared to the control group (P = 0.0001). Compared with the control group, portal vein wet weight and PVT length were significantly decreased in the experimental group of rats at 1 day after modeling (P < 0.05), whereas these differences were not found in the two groups of rats at 3 and 5 days after modeling (P > 0.05). The percentage of non-organized thrombus area in the experimental group was significantly decreased compared with that in the control group at 1, 3, and 5 days after modeling (P < 0.05), whereas there was no significant difference in the percentage of lumen area of organized thrombus between the two groups (P > 0.05).

**Conclusion:**

The method of producing a rat PVT model by destroying the endothelium of the portal vein by anhydrous ethanol combined with blood flow stasis is feasible and reproducible. In addition, the optimal time window for thrombolysis in the treatment of PVT in rats using urokinase is the early stage of thrombosis, when the fibrin content is highest.

## Introduction

Portal vein thrombosis (PVT) is a blood clot that obstructs the main lumen of the portal vein and may extend to intrahepatic or extrahepatic venous branches [1]. PVT is associated with various conditions, including cirrhosis, portal hypertension, malignant tumors, abdominal infections, and the sequelae of abdominal surgery [2]. As a common complication of cirrhosis, the incidence of PVT has gradually increased in recent years. The prevalence of PVT in cirrhotic patients with Child classifications B and C is 17% [3]. PVT is a condition with multifactorial pathogenesis, gradual onset, and subtle clinical manifestations. It can result in severe complications, including liver damage, ischemic intestinal necrosis, and varicose vein hemorrhage [4]. Severe PVT results in hepatic decompensation as evidenced by decreased plasma albumin(ALB) levels, and elevated total bilirubin(TBil) levels. Therefore, prompt and effective treatment of PVT is of utmost importance. The treatment for portal vein thrombosis involves anticoagulation and thrombolysis [5]. Anticoagulation may increase the rate of portal vein recanalization without significantly increasing the risk of bleeding [6, 7]. However, anticoagulation therapy still requires frequent dosing adjustments due to the common complications of cirrhosis, such as renal insufficiency, ascites, and edema [8]. Furthermore, although thrombolysis is crucial in the management of acute PVT [9], its specific time window for thrombolysis remains uncertain.

Current thrombolytic drugs mainly include urokinase, tissue-type plasminogen activator and recombinant tissue-type plasminogen activator. Urokinase acts on the endogenous fibrinolytic system to promote the production of fibrinolytic enzymes. The dissolution of fibrin is enhanced by fibrinolytic enzymes, resulting in prolongation of prothrombin time (PT), a decrease in fibrinogen(FBG) levels and an increase in fibrin degradation products levels, such as D-dimers(D2D) [10]. Currently, urokinase has been used in the treatment of acute cardiac and cerebral stroke [11]. However, there is no consensus on its use for PVT. Reasons for this may include the scarcity of specimens due to the specialized physiology of the portal vein and the difficulty of conducting direct clinical studies of thrombolytic therapy with urokinase.

Currently, there are three studies describing the production of rat PVT models. Zhang et al. [12] constructed a model by intermittently ligating the distal and proximal portal veins of rats to block blood flow. On this basis, Yao [13] and Wei et al. [14] added clamping of blood vessels. They initiated the coagulation system by enhancing vascular endothelial cell injury, which induced PVT formation. The site of thrombus formation in these methods is relatively fixed and easy to observe. However, uncontrollable factors such as ligation strength and degree of endothelial damage make PVT formation unstable.

In the early stages of thrombosis, the main component of fresh thrombus is a large accumulation of red blood cells (non-organized thrombus). As time goes by, endothelial cells and fibroblasts gradually grow out of the vessel wall, and granulation tissue is formed. Granulation tissue extends into thrombus, gradually forming organized thrombus. Currently, there are no studies focused on observing the developmental stage of PVT organization [15]. Therefore, we developed a stable rat PVT model and explored the process of PVT formation and thrombus organization. Further, urokinase thrombolysis was performed for PVTs with different degrees of thrombus organization. Our aim was to provide a time window for the clinical application of urokinase in the treatment of PVT.

## Materials and methods

### Chemical reagent

Rat Fibrinogen ELISA Kit (Quanzhou Ruixin Biological Technology Co., Ltd. Quanzhou, China); Rat D-dimer ELISA Kit (Quanzhou Ruixin Biological Technology Co., Ltd. Quanzhou, China); Rat prothrombin time ELISA Kit (Quanzhou Ruixin Biological Technology Co., Ltd. Quanzhou, China); Rat albumin ELISA Kit (Quanzhou Ruixin Biological Technology Co., Ltd. Quanzhou, China); Rat total bilirubin Kit (Quanzhou Ruixin Biological Technology Co., Ltd. Quanzhou, China); Sevoflurane for inhalation (Shanghai Hengrui Medicine Co., Ltd. Shanghai, China); Anhydrous ethanol (Shandong Lierkang Medical Technology Co., Ltd, Shandong, China); EDTA-K2 vacuum blood collection tubes (Tianjin Biochemical Pharmaceutical Co., Ltd, Tianjin, China); Urokinase (Tianjin Biochemical Pharmaceutical Co., Ltd, Tianjin, China); Rat CD31 immunohistochemical antibody (Quanzhou Ruixin Biological Technology Co., Ltd. Quanzhou, China).

### Animals

Fifty-one clean-grade male Sprague-Dawley (SD) rats, 8–10 weeks old, weighing 350 ± 50 g. All experimental procedures and animal housing were approved by the Animal Protection and Use Committee of Chengde Medical College (Protocol Number: CYFYLL2021210). All surgery was performed under sevoflurane anesthesia, and all efforts were made to minimize suffering.

### Rat grouping

After successfully modeling the rat PVT, forty-eight rats were randomly divided into two groups: an experimental group (urokinase treatment group) and a control group (saline group), with 24 rats in each group. And 8 rats were randomly selected from the two groups to be dissected at 1, 3 and 5 days after modeling, respectively. There were no significant differences in weight between groups of rats. Changes in specimen plasma FBG, D2D, and PT levels were assured to be measurable by injecting urokinase 24h and 12h before autopsy. The dose of urokinase administered to rats was converted according to U.S. Food and Drug Administration standards. Rats in the experimental group were injected with urokinase (50,000 μ/kg*2) in the tail vein 24h and 12h before autopsy, respectively; rats in the control group were injected with saline at the same time and in the same way. Three healthy rats were used to observe the pathologic organization of normal portal vein thrombosis.

### Establishment of rat PVT model

The rat PVT model was produced by combining anhydrous ethanol disruption of portal endothelium with stasis of blood flow. Rats were anesthetized with sevoflurane inhalation followed by abdominal disinfection with iodophor. A median abdominal incision was made to visualize the portal vein. Microvascular clips were used to block the distal and proximal portals of the portal vein (The proximal end is the bifurcation of the left and right branches of the liver, and the distal end is level with the splenic vein). In order to disrupt the endothelium of the portal vein, anhydrous ethanol was injected into the portal vein for aspiration lasting 60s. Then, the distal vascular clamp was released to allow the portal vein to fill, while a cotton swab was used to compress the needle hole to stop bleeding. After successful hemostasis, the distal vascular clamp was closed again to block the portal blood flow for 2 min. Thrombus formation was visible to the naked eye after the vascular clamps were released. Further observe the intestinal blood flow status. If the color of the intestinal wall changes from light blue to red indicates that the intestinal blood flow status has recovered well, and the abdominal cavity can be closed to end the modeling.

### Acquisition of PVT organization

PVT tissue samples were collected from control and experimental rats at 1, 3 and 5 days after modeling. Rats were anesthetized with sevoflurane, and a midline abdominal incision was made after abdominal sterilization. The portal vein was exposed, and then the section between the right and left hepatic bifurcation points and the splenic vein was resected. Finally, thrombus length and portal vein wet weight were measured. Rats were sacrificed by cervical dislocation after obtaining specimens under anesthesia.

### Measurement of plasma FBG, D2D, PT, ALB and TBil levels

EDTA-K2 vacuum blood collection tubes were used to collect 2 ml of blood from the inferior vena cava and centrifuged at 4000 rpm/min for 20 min at 4°C. Take the upper layer of plasma to be divided and labeled. Medical cryogenic refrigerator (-80°C) was used to store the specimens centrally. After all samples were collected, rat plasma FBG, D2D, PT and ALB levels were measured using ELISA kits according to the instruction manual. Plasma TBil levels in rats were measured by the chemical oxidation method according to the kit instructions.

### Immunohistochemistry

Immunohistochemistry using 4-μm-thick PVT sections. Sections were deparaffinized and rehydrated for subsequent heat-mediated antigen repair. Goat serum was applied to adequately seal the sections and block endogenous peroxidase. Sections titrated with CD31 antibody were incubated at 4°C overnight. Sections were incubated with goat anti-mouse secondary antibody for 1h at room temperature. Diaminobenzidine was used for tissue color development and hematoxylin was used to re-stain the sections. Cover slips were covered after re-staining. The stained sections were visualized by microscopy. Four images at 400 x magnification were selected for each pathological tissue and utilized to enumerate the number of endothelial cells in the inner wall of the portal vein.

### Measurement of the degree of occlusion of the portal vein lumen

The thrombus tissues were processed for HE staining and Masson trichrome staining after sequential fixation, dehydration, transparency, paraffin embedding, sectioning, and deparaffinization, respectively. ImageJ software was used to measure thrombus as a proportion of portal vein lumen area.

### Statistical analysis

The statistical analysis was performed using GraphPad Prism software (version:8.0.2). Experimental data were described as mean ± standard error. The data between multiple groups was compared using one-way ANOVA, and Tukey’s multiple comparisons test was used to analyze the data related to each indicator between the control groups. Unpaired t-test was chosen for comparison of the two groups of data. A P value *<*0.05 was considered to indicate statistical significance.

## Results

### Rat portal vein thrombosis modeling

**Fig 1:**
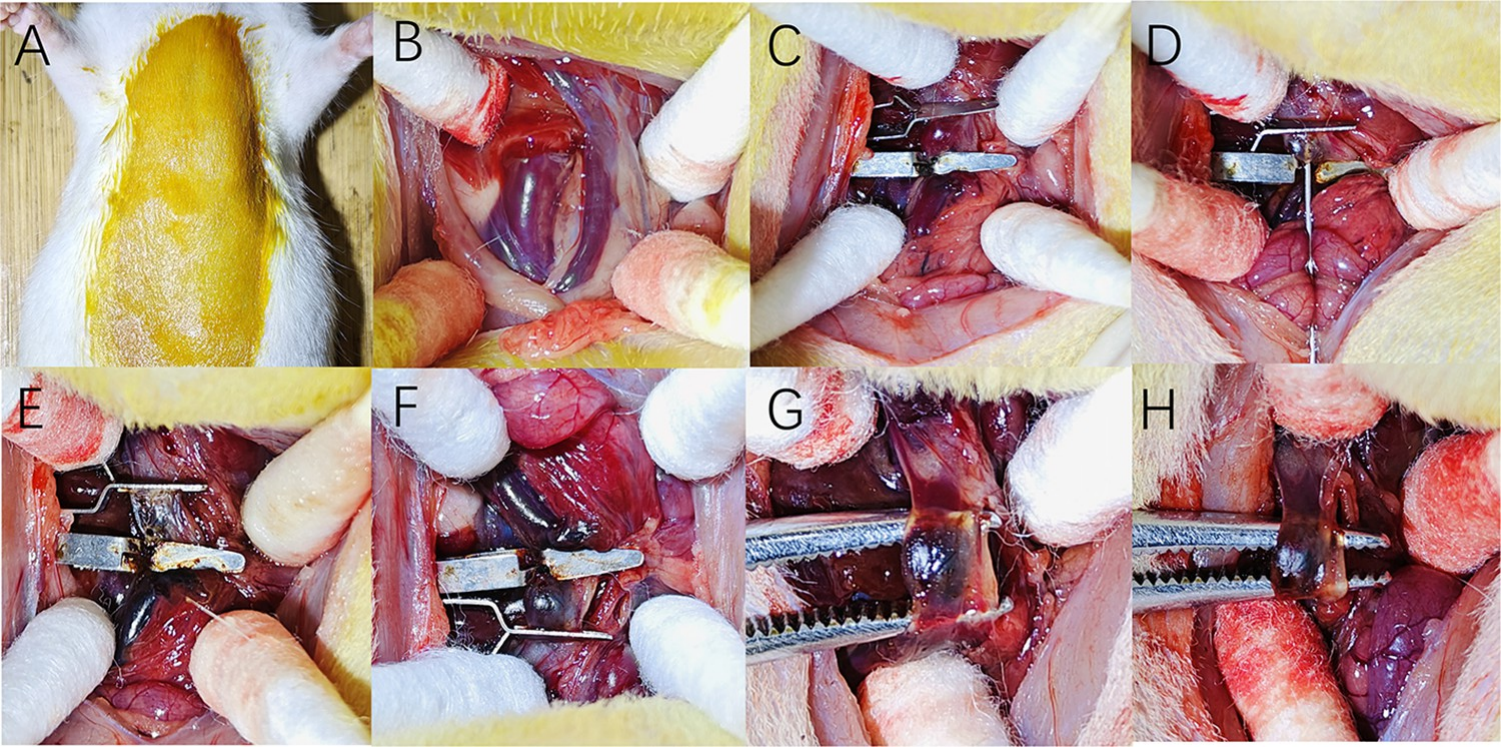
The procedure of rat PVT modeling. A: Skin disinfection of rats before modeling; B: Exposing the portal vein (inferior vena cava on the left and portal vein on the right); C: Closing the portal vein using vascular clamps. D: Injection of anhydrous ethanol into the portal vein for aspiration; E: Macroscopic view of the portal vein after anhydrous ethanol injection; F: portal vein stasis after anhydrous ethanol injection; G-H: Macroscopic view of the formation of PVT after stasis.

The specific process of rat PVT modeling is shown in Fig 1.

### Disruption of portal vein endothelium after modeling

**Fig 2:**
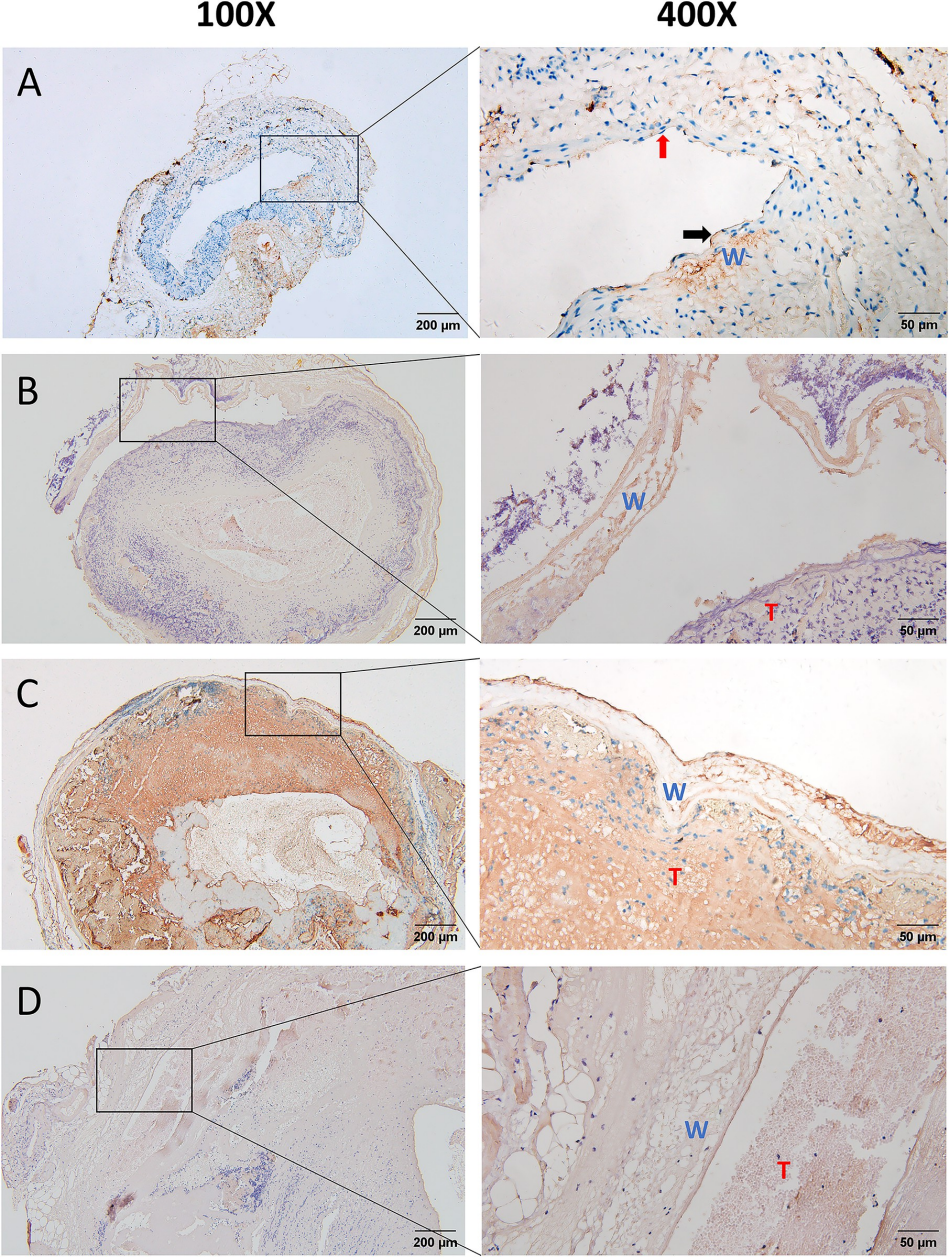
CD31 immunohistochemical staining of portal vein in each group of rats. A: CD31 immunohistochemistry staining of portal vein in healthy rats. Black arrows point to complete arrangement of endothelial cells; red arrows point to endothelial cell nuclei. B/C/D: CD31 immunohistochemical staining of portal vein in rats 1/3/5 days after modeling. W indicates portal vein wall; T indicates thrombus.

The success and mortality rates of the rat PVT model used in this study we have previously reported [16]. To determine whether anhydrous ethanol caused damage to the portal vein endothelium, we performed immunohistochemistry to observe CD31 expression in the portal vein endothelium. As shown in Fig 2A, continuous high expression of CD31 and complete arrangement of endothelial cells were seen on the inner side of the portal vein wall in healthy rats. However, the continuous high expression of CD31 in the rat portal vein inner wall on days 1, 3, and 5 after modeling was not observed (Fig 2B–2D). In addition, three healthy rats and nine control rats (three each on days 1, 3, and 5 after modeling) were selected to count the number of endothelial cells. The number of endothelial cells was found to be significantly decreased in rats at 1, 3, and 5 days after modeling compared to healthy rats(33.67±2.56 vs 1.08±0.41 pcs, P < 0.0001; 33.67±2.56 vs 1.17±0.37 pcs, P < 0.0001 and 33.67±2.56 vs 1.50±0.53 pcs, P < 0.0001)(S1 Fig). In addition, no significant difference in the number of endothelial cells was observed between the three groups of rats at 1, 3, and 5 days after modeling(1.08±0.41 vs 1.17±0.37 pcs, P = 0.88; 1.08±0.41 vs 1.50±0.53 pcs, P = 0.54 and 1.17±0.37 vs 1.50±0.53 pcs, P = 0.61)(S1 Fig). These results suggest that anhydrous ethanol did disrupt the portal vein endothelium during modeling.

### Portal vein wet weight and thrombus length in control rats after modeling

Portal vein wet weight and thrombus length were measured separately after obtaining PVT specimens from control rats. As depicted in S2A Fig, the wet weights of the rat portal vein at 1, 3, and 5 days after modeling surgery were 34.61±1.47 mg, 31.13±2.34 mg, and 32.56±2.00 mg, respectively, which did not exhibit significant differences with the time of PVT formation (34.61±1.47 vs 31.13±2.34 mg, P = 0.44; 34.61±1.47 vs 32.56±2.00 mg, P = 0.75 and 31.13±2.34 vs 32.56±2.00 mg, P = 0.86). The thrombus lengths of rats at 1, 3, and 5 days after modeling were 4.81±0.23 mm, 4.24±0.32 mm, and 4.69±0.26 mm, respectively (S2B Fig). There were no significant differences in thrombus lengths of control rats at the three time points (4.81±0.23 vs 4.24±0.32 mm, P = 0.31; 4.81±0.23 vs 4.69±0.26 mm, P = 0.94 and 4.24±0.32 vs 4.69±0.26 mm, P = 0.48).

### Degree of portal vein occlusion in control rats after modeling

**Fig 3:**
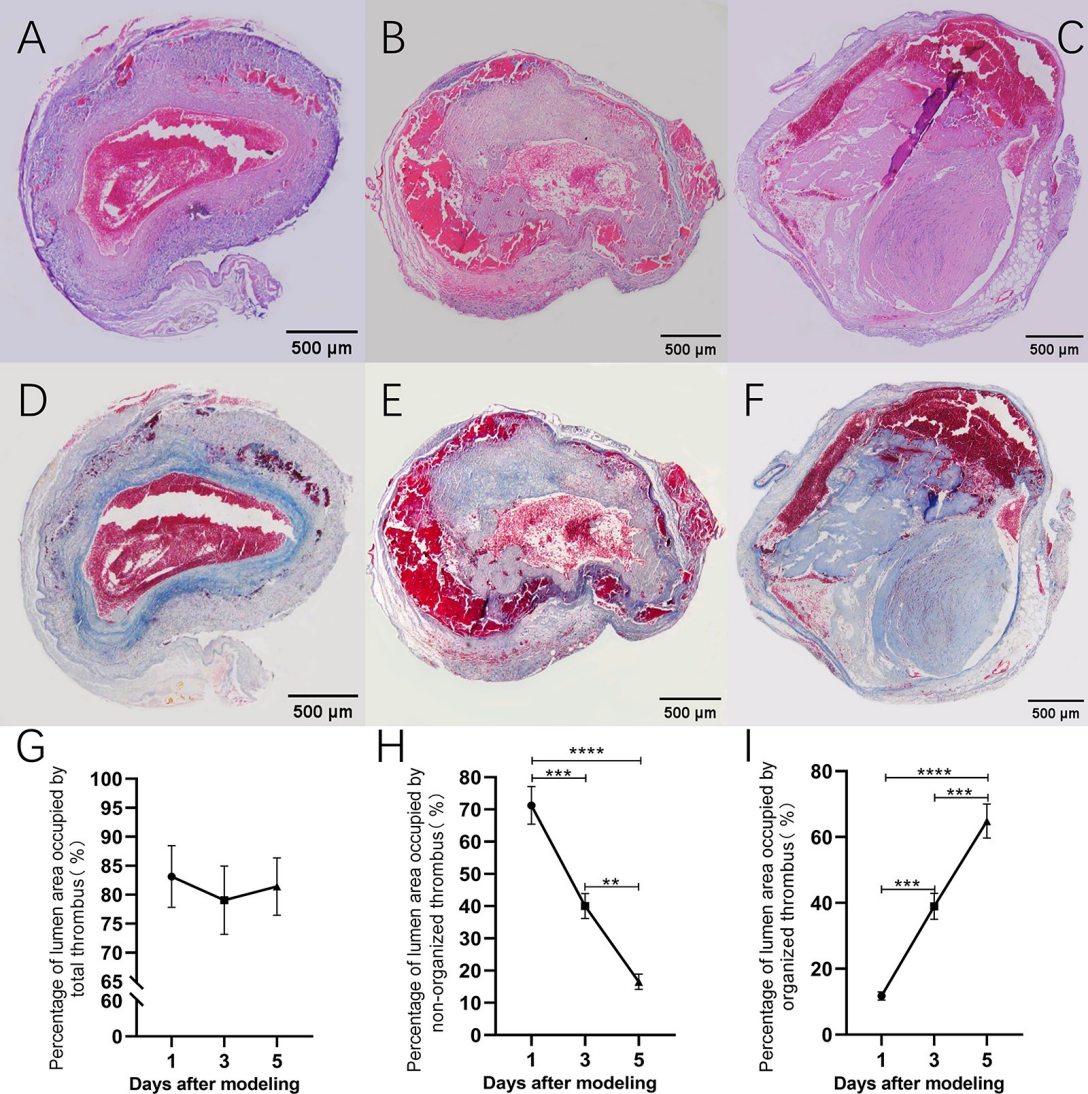
HE staining (A/B/C) and Masson staining (D/E/F) of portal vein in control rats 1/3/5 days after modeling; G: Percentage of luminal area of total thrombus in the portal vein 1/3/5 days after modeling in control rats. H: Percentage of luminal area of non-organized thrombus in the portal vein 1/3/5 days after modeling in control rats; I: Percentage of luminal area of organized thrombus in the portal vein 1/3/5 days after modeling in control rats.

HE and Masson staining of PVT in control rats is shown in Fig 3A–3F. The degree of portal lumen occlusion in rats was evaluated by observing HE-stained sections of thrombus tissue from control rats (Fig 3A–3C). As shown in Fig 3G, the percentage of portal vein lumen occlusion area was 83.13±5.33%, 79.02±5.90%, and 81.39±4.94% on days 1, 3, and 5 after modeling in rats, respectively. There was no significant difference in the degree of portal vein lumen occlusion in rats at the three time points after modeling (83.13±5.33 vs 79.02±5.90%, p = 0.85; 83.13±5.33 vs 81.39±4.94%, p = 0.97 and 79.02±5.90 VS 81.39±4.94%, p = 0.95). Upon further observation of thrombus composition, it was found that the composition of rat PVT was dominated by erythrocytes one day after modeling (Fig 3A). Fibrin exudation was observed at three days after modeling (Fig 3B), and a significant amount of fibrin infiltration was seen at five days after modeling (Fig 3C). These findings indicate that the rat PVT was progressively organized with the time of formation.

### Degree of rat PVT organization after modeling

Masson staining was used to assess the degree of rat PVT organization after modeling (Fig 3D–3F). After successful modeling, the percentage of luminal area of non-organized thrombus in the rat portal vein showed a decreasing trend with time (Fig 3H). The percentage of non-organized thrombus lumen area was significantly reduced at 3 and 5 days after modeling compared with 1 day after modeling (71.27.27±5.83 vs. 40.05±3.86%, P = 0.0001 and 71.27±5.83 vs. 16.53±2.34%, P < 0.0001, respectively); A significant decrease in the percentage of non-organized thrombus lumen area was also observed when comparing 3 and 5 days after modeling (40.05±3.86 vs 16.53±2.34%, P<0.01).

As shown in Fig 3I, the percentage of luminal area of organized thrombus in the rat portal vein tended to increase over time. The percentage of luminal area of organized thrombus in the portal vein was significantly higher in rats at 3 and 5 days after modeling compared with 1 day after modeling (11.86±1.22 vs 38.97±3.95%, P<0.001 and 11.86±1.22 vs 64.86±5.18%, P<0.0001). A significant increase in PVT organization was also observed when comparing 3 and 5 days after modeling (38.97 ± 3.95 vs. 64.86 ± 5.18%, P < 0.001).

### Efficacy of urokinase on PVT in rats

**Fig 4:**
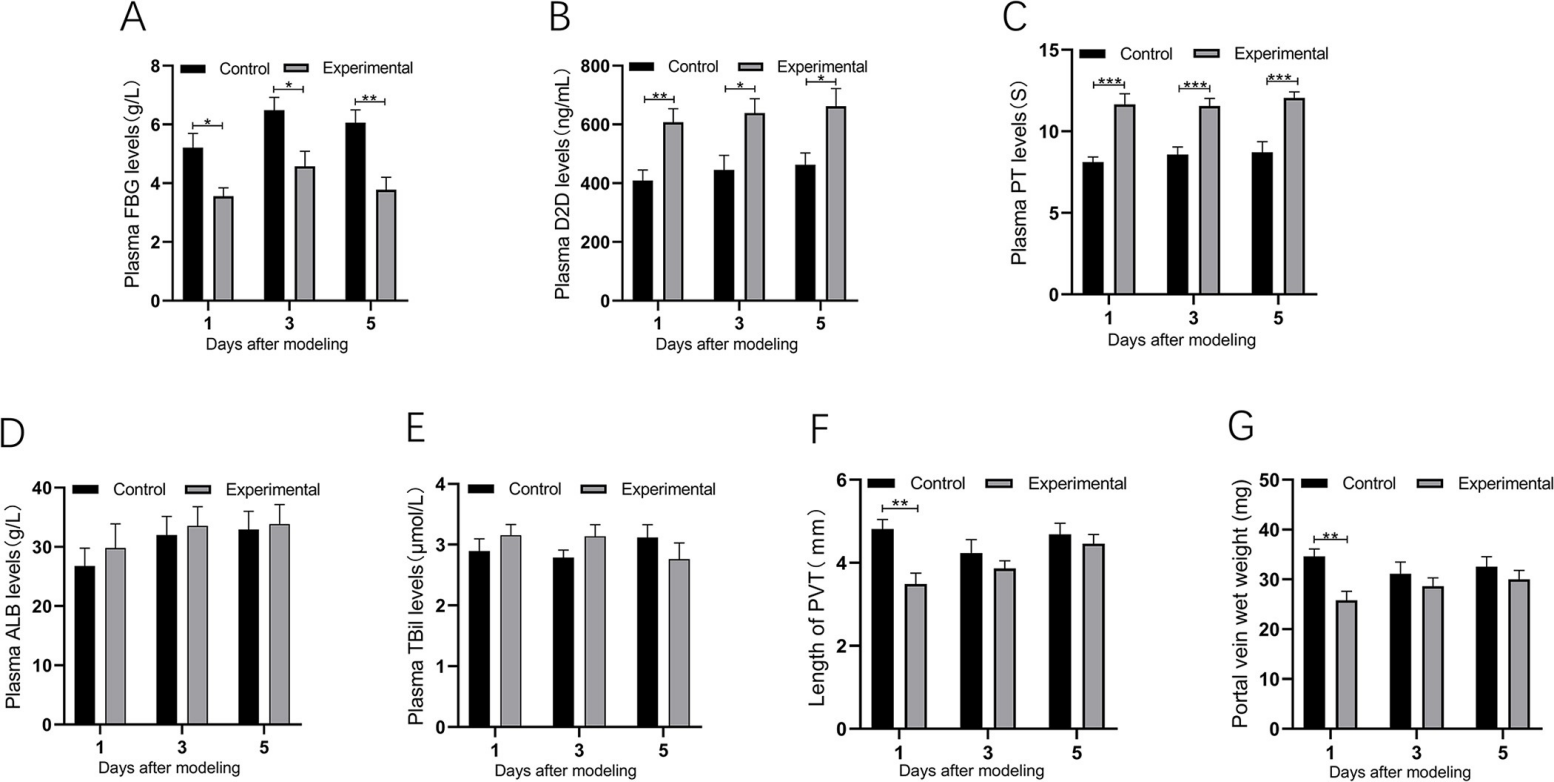
A-E: Comparison of plasma fibrinogen (A), D-dimer (B), prothrombin time (C), albumin(D) and total bilirubin(E) levels in control and experimental rats; F and G: Comparison of portal vein wet weight (F) and thrombus length (G) in control and experimental rats.

To confirm the effectiveness of the urokinase dose, we measured the levels of plasma fibrinogen (FBG) and its degradation product D-dimer (D2D) in rats. As shown in Fig 4A, plasma FBG levels were significantly lower in urokinase-treated experimental rats compared with control rats at 1, 3, and 5 days after modeling (5.21±0.48 vs. 3.56±0.28 g/L, P<0.05; 6.49±0.43 vs. 4.58±0.51 g/L, P<0.05 and 6.06±0.44 vs. 3.77 ± 0.43 g/L, P < 0.01); Plasma D2D levels were significantly higher in the experimental group of rats compared to the control group at 1, 3 and 5 days after modeling (409.03±36.29 vs. 607.92±45.58 ng/mL, P<0.01; 445.60±49.33 vs. 639.09±48.51 ng/mL, P<0.05 and 463.10±40.12 vs. 662.30 ± 60.05 ng/mL, P < 0.05) (Fig 4B).

We observed changes in rat coagulation function, with significantly prolonged PT in the experimental group compared with the control group at 1, 3, and 5 days after modeling (8.12±0.32 vs. 11.66±0.64 ng/mL, P = 0.0002; 8.58±0.47 vs. 11.55±0.46 ng/mL, P = 0.0005 and 8.47±0.57 vs 12.05±0.37ng/mL, P = 0.0001) (Fig 4C).

In addition, we measured plasma ALB and TBil levels in both groups of rats in order to observe changes in liver function. No significant differences in plasma ALB levels were found between experimental and control rats at 1, 3, and 5 days after modeling(31.34±3.33 vs 26.79±3.00g/L, P = 0.33; 29.81±4.07 vs 32.00±3.15g/L, P = 0.68 and 33.84±3.32 vs 32.94±3.07g/L, P = 0.85) (Fig 4D). In addition, no significant differences in plasma TBil levels were found between experimental and control rats at 1, 3, and 5 days after modeling(3.16±0.18 vs. 2.97±0.22μmol/L, P = 0.52; 3.14±0.19 vs 2.79±0.12μmol/L, P = 0.14 and 2.76±0.27 vs 3.12±0.21μmol/L, P = 0.31) (Fig 4E). These results indicated that the use of urokinase did not lead to impairment of the liver function.

### Treatment with urokinase immediately after PVT formation reduces portal wet weight and thrombus length

As shown in Fig 4F, portal vein wet weight was significantly lower in the experimental group of rats 1 day after modeling compared with control rats (34.61 ± 1.47 vs. 24.81 ± 1.79 mg, P < 0.01). However, comparing experimental and control rats at 3 and 5 days after modeling, no significant difference in portal vein wet weight was found (31.13 ± 2.34 vs 28.13 ± 2.67 mg, P = 0.40 and 32.56 ± 2.00 vs 30.01 ± 1.80 mg, P = 0.36).

As shown in Fig 4G, treatment with urokinase immediately after PVT formation reduced thrombus length, which was significantly shorter in the experimental group of rats at 1 day after modeling compared with control rats (4.81 ± 0.23 mm vs. 3.49 ± 0.26 mm, P < 0.01). No significant difference in thrombus length was observed between experimental and control rats at 3 and 5 days after modeling (4.24 ± 0.32 vs 3.86 ± 0.18 mg, P = 0.32 and 4.69 ± 0.26 vs 4.46 ± 0.22 mg, P = 0.52, respectively).

### Urokinase reduces portal lumen occlusion by dissolving non-organized thrombi

**Fig 5:**
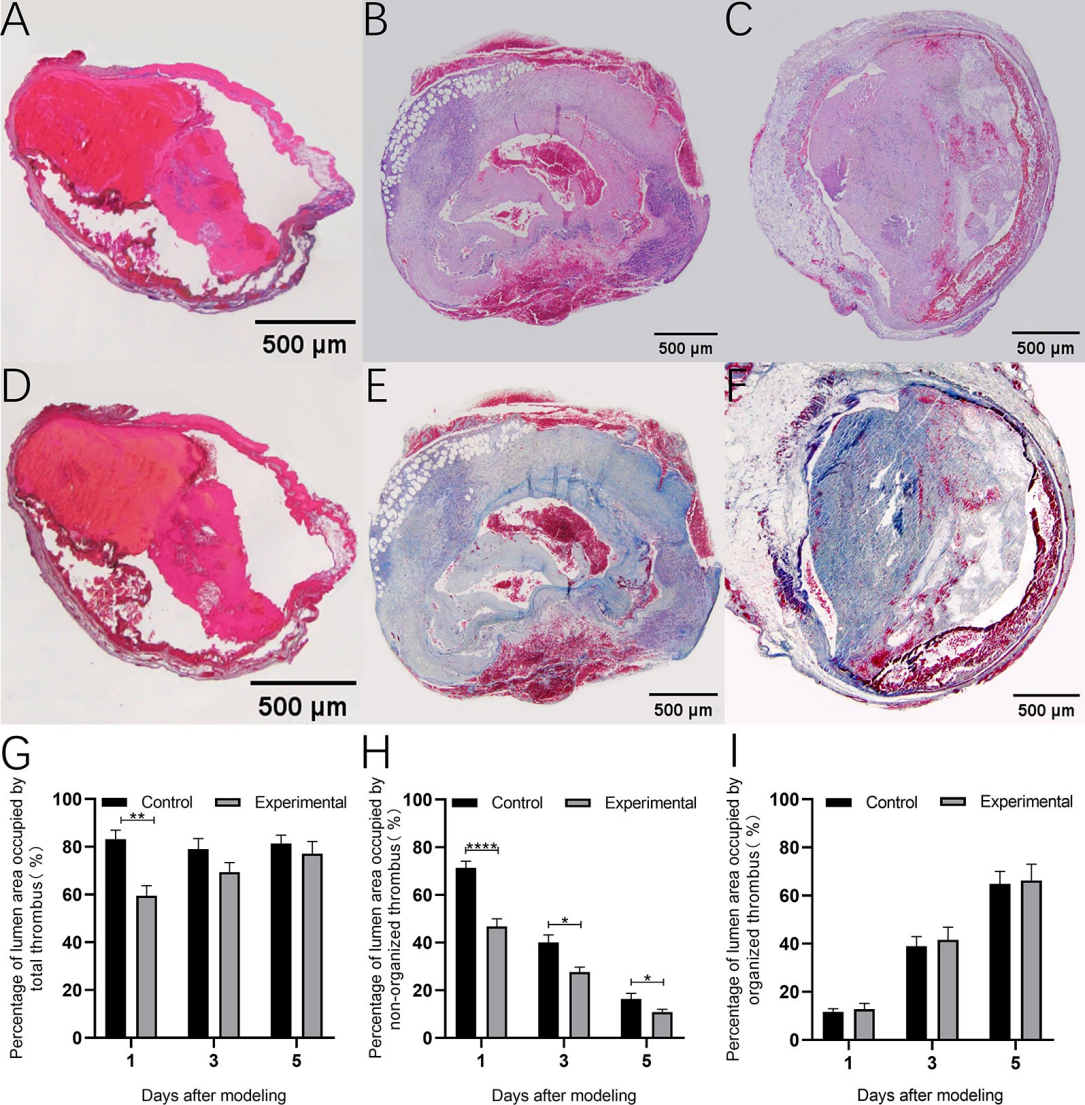
HE staining (A/B/C) and Masson staining (D/E/F) of portal vein in Experimental rats 1/3/5 days after modeling; G: Comparison of the percentage of lumen area occupied by total thrombus between control and experimental rats after 1/3/5 days of modeling; H: Comparison of the percentage of lumen area occupied by non-organized thrombus between control and experimental rats after 1/3/5 days of modeling; I: Comparison of the percentage of lumen area occupied by organized thrombus between control and experimental rats after 1/3/5 days of modeling.

HE and Masson staining of PVT in the experimental group of rats is shown in Fig 5A–5F. Treatment with urokinase immediately after PVT formation reduces the degree of occlusion of the portal vein lumen. As shown in Fig 5G, the percentage of luminal occlusion area was significantly reduced in the experimental group of rats at 1 day after modeling compared with the control group (83.13 ± 5.33 vs 59.57 ± 4.11%, P < 0.01). However, no significant difference in the percentage of area of portal vein lumen occlusion was observed between experimental and control rats at 3 and 5 days after modeling (79.02 ± 5.90 vs 69.38 ± 4.02%, P = 0.20 and 81.39 ± 4.94 vs 77.12 ± 5.11%, P = 0.56, respectively).

Further comparison of the percentage of luminal area in the portal vein between non-organized thrombus and organized thrombus. As shown in Fig 5H, after urokinase treatment, the lumen percentage of non-organized thrombus area in the experimental group was significantly reduced compared with that in the control group at 1, 3, and 5 days after modeling (71.27 ± 5.83 vs. 46.81 ± 3.18%, P < 0.0001; 40.05 ± 3.86 vs. 26.37 ± 2.98%, P < 0.05 and 16.53 ± 2.34 vs. 10.87 ± 1.15%, P < 0.05); Whereas, the percentage of luminal area of organized thrombus was not significantly different between the two groups of rats at 1, 3, and 5 days postoperatively (11.86 ± 1.22 vs. 12.75 ± 2.42%, P = 0.80; 38.97 ± 3.95 vs. 41.63 ± 5.20%, P = 0.72 and 64.86 ± 5.18 vs. 66.25 ± 6.28%, P = 0.89, respectively) (Fig 5I). These findings indicate that urokinase reduces the area of portal lumen occlusion by dissolving non-organized thrombi.

## Discussion

PVT is a type of venous thrombosis. The severity of symptoms is related to the cause and extent of the PVT. Although most PVT are clinically asymptomatic, they have the potential to cause bowel ischemia and infarction when the thrombus involves the superior mesenteric vein, with an associated mortality rate of up to 60% [17]. The pathophysiological mechanism of PVT may differ from that of deep vein thrombosis due to the unique closed anatomy of the portal venous system and the absence of venous valves in the portal vein [18]. By analyzing existing studies, we found that the pathophysiological mechanisms and therapeutic regimens of PVT have not been adequately described due to the difficulty in obtaining large numbers of PVT specimens in the clinic and the relative lack of relevant animal models [19]. Therefore, stable and reproducible animal models of PVT are essential.

Previous studies have demonstrated the feasibility of constructing PVT models in other animals. Deng et al. [20] developed a model of PVT in dogs by catheterizing the portal vein for thrombin injection. This method necessitates a high puncture base for the operator and the catheter is prone to clogging. Furthermore, this method requires continuous injection of antibiotic therapy after modeling, which tends to impede the accuracy of subsequent PVT-related studies. Zhang et al. [21] employed ultrasound-guided catheter puncture for thrombin injection to create a swine PVT model. However, they also acknowledged that the small sample size may have affected the accuracy of the results. In fact, the production of PVT models of large-sized animals on a large scale is challenging. The rat was selected as the subject for the PVT model due to the relative simplicity of the modeling method. Consequently, the rat PVT model can be mass-produced, thereby reducing the potential for bias in the accuracy of the results due to a small sample size.

This article provides a stable and reproducible animal model of PVT by blood stasis combined with endothelial disruption, and anatomical observations were conducted at 1, 3, and 5 days after modeling. No significant differences were observed in PVT weight and thrombus length in rats within 5 days of PVT formation. Upon closer observation, we found that the proportion of thrombus components changed, although there was no significant difference in the degree of portal vein occlusion within 5 days after successful modeling. On the first day after modeling, rats formed a nearly full lumen of fresh thrombus with a predominantly erythrocytic composition. Subsequently, fresh thrombus was converted to organizing thrombus, and a significantly higher percentage of luminal area of organizing thrombus was found at both day 3 and day 5 after modeling compared to day 1 after modeling. The basic pathological process of thrombus organization is the gradual replacement of the thrombus mass in the lumen by granulation tissue generated in the vessel wall [15]. We compared the number of endothelial cells in the inner wall at 1, 3 and 5 days after modeling and found no significant difference between the three time points. However, the degree of PVT organization was observed to increase at 1, 3, and 5 days after modeling. Consequently, we postulated that the PVT became progressively organised with the passage of time, whereas endothelial disruption was more likely to result in the formation of PVT than to the development of PVT organization.

In recent years, the gradual development and popularization of endovascular selective catheter thrombolysis has made thrombolysis for acute PVT more feasible [19]. A meta-analysis by Gao et al. showed that the overall response rate for urokinase treatment of PVT was 93% by analyzing 29 relevant studies. Compared to thrombolytic therapy given within 14 days of symptom onset, the overall response rate for PVT treated with urokinase for >14 days of symptom onset was significantly lower [22]; In addition, they reported a detailed treatment protocol for a patient with PVT who successfully relieved PVT symptoms after thrombolytic therapy combined with anticoagulation [23]. Jiang et al. [9] showed that 20 patients with acute PVT were treated by transcatheter superior mesenteric artery urokinase infusion, and 75% of them showed improvement of PVT symptoms and maintained continuous recanalization for the next 6 months. Although thrombolysis has shown initial success in the treatment of acute PVT, there is no consensus on the time window for thrombolysis in PVT.

This article explores the urokinase thrombolytic time window by administering urokinase therapy at different time points after PVT formation. In comparison to the control rats, the experimental rats had increased plasma FBG levels and decreased D2D levels, which was caused by the injection of urokinase 24h and 12h prior to the collection of blood samples in the experimental group. The pathological picture demonstrated that thrombolytic therapy with urokinase immediately after thrombosis significantly reduced the degree of portal vein obstruction. It may be that the nature of fresh thrombus formed after rat modeling is aggregation of erythrocytes with fibrin as a network, and urokinase contributes to fibrin degradation by acting on the endogenous fibrinolytic system, resulting in PVT dissolution. Treatment with urokinase at 2 and 4 days after modeling did not result in a significant reduction in the degree of portal vein occlusion. This may be due to partial thrombus mechanization resulting in a lower percentage of fresh thrombus that can be dissolved by urokinase, and therefore the efficacy of urokinase is reduced.

The time window for urokinase thrombolysis in stroke has been widely studied [24]. However, studies on urokinase thrombolysis for PVT are less common. Currently, PVT in clinical practice tends to have a chronic course and is generally accompanied by cirrhosis. Driever et al. [25] have analyzed the composition and structure of PVT in 63 patients with cirrhosis. The results showed that fibrin-rich PVT was found in only one-third of the patients, which is why not all patients with PVT can be recanalized by thrombolytic therapy. In this study, the fibrin content of the thrombus was highest in the early stages of PVT formation, and treatment with urokinase thrombolysis given at this time significantly reduced luminal occlusion. However, when the degree of thrombus organization increased, the thrombolytic effect of urokinase was greatly reduced. In addition, whether the pathophysiologic mechanisms underlying the development and progression of PVT correlate with its venous wall level still needs to be further explored. However, this study has some limitations. Although we have provided the optimal time window for urokinase thrombolysis for the treatment of PVT in rats, further validation is needed for the application of this time window in clinical practice. In addition, the diameter of the portal vein is smaller in rats than in humans, which may affect the accuracy of the results.

Overall, the method of creating a rat portal vein thrombosis model by combining disruption of the portal vein endothelium with blood stasis is feasible and reproducible, and it complements the relatively empty field of PVT modeling; In addition, the optimal time window for thrombolysis in the treatment of PVT in rats using urokinase is the early stage of thrombosis, when the fibrin content is highest.

## Supporting information

S1 Fig Number of endothelial cells in the inner wall of the portal vein in each group of rats. (TIF)

S2 Fig Portal vein wet weight and thrombus length in control rats. (TIF)

S1 Data (PZFX)
