# Supplementary figures and images for: Model construction and thrombolytic treatment of rat portal vein thrombosis
Source: PLoS One. 2024 Aug 2;19(8):e0308178. doi: 10.1371/journal.pone.0308178 (PMC11296622; doi:10.1371/journal.pone.0308178)

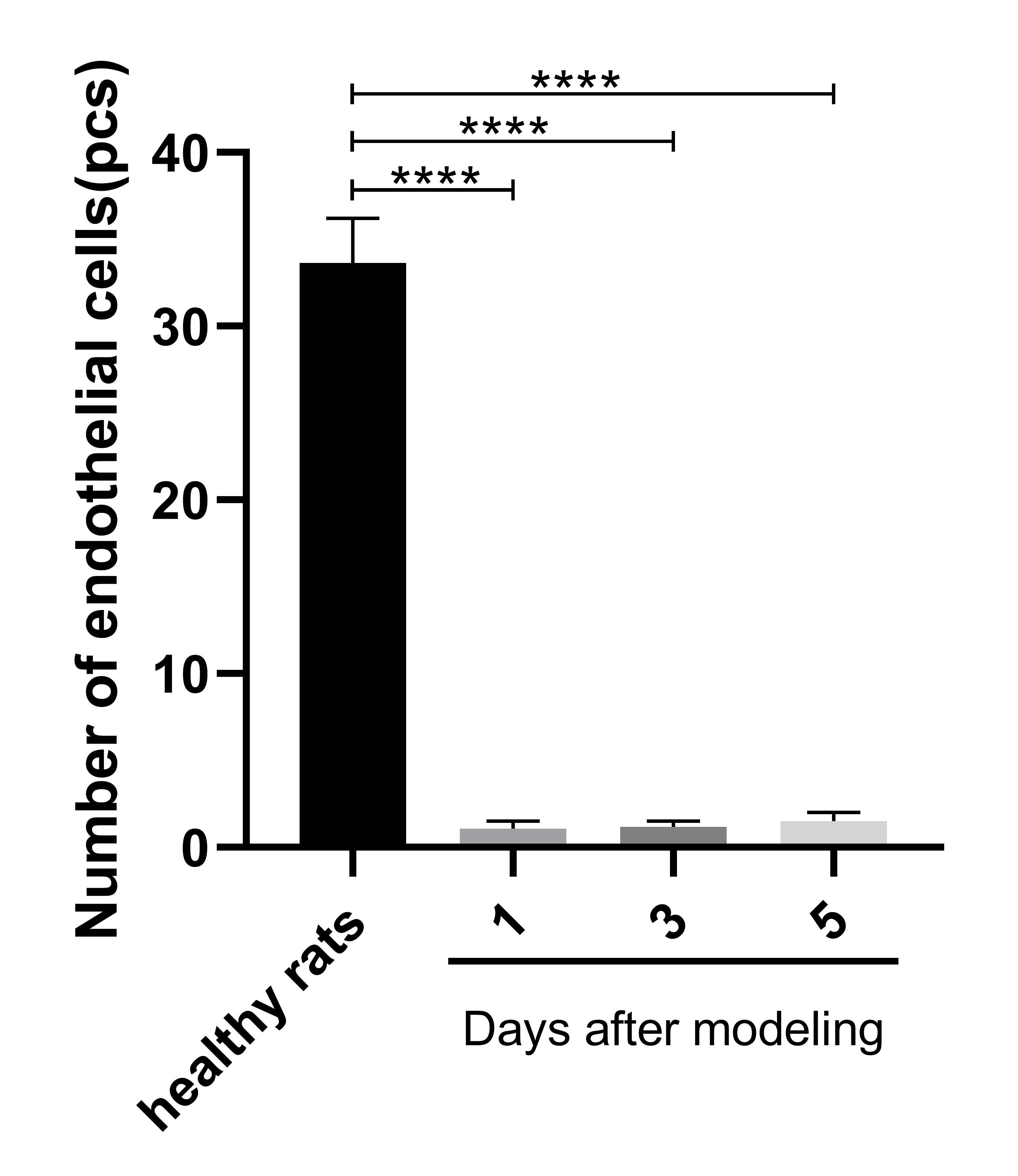

Supplement: S1 Fig — (TIF) [file pone.0308178.s001.tif]

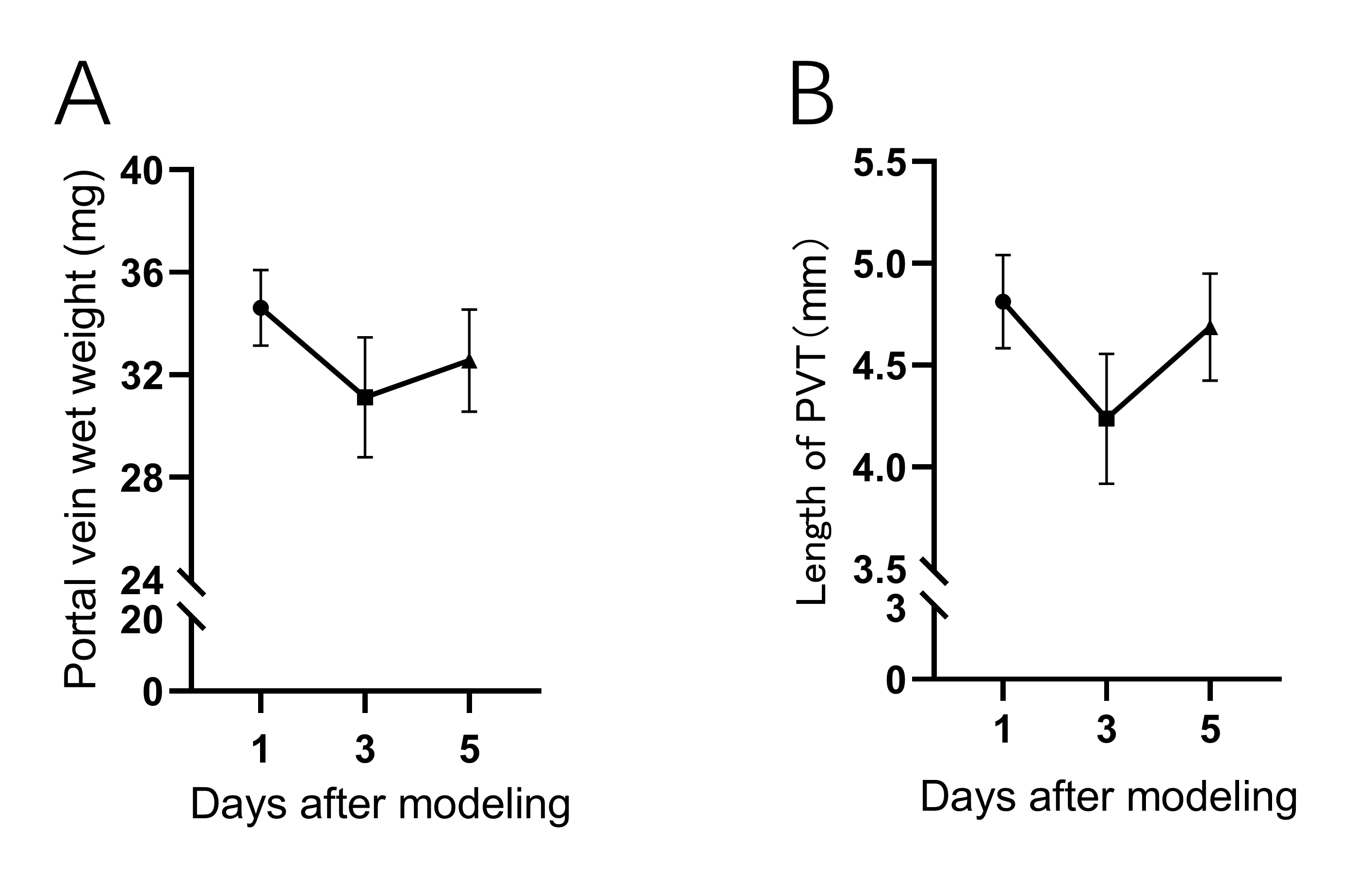

Supplement: S2 Fig — (TIF) [file pone.0308178.s002.tif]
